# Supplementary material for: Pilates versus resistance training on trunk strength and balance adaptations in older women: a randomized controlled trial
Source: PeerJ. 2019 Nov 14;7:e7948. doi: 10.7717/peerj.7948 (PMC6859004; doi:10.7717/peerj.7948)
Supplement: Supplemental Information 3 [file peerj-07-7948-s003.docx]

| Table S1. Examples of Pilates exercises. | | | |
| --- | --- | --- | --- |
| MESOCYCLE | EXERCISE FOCUSHED ON THE SPINE | EXERCISE FOCUSHED ON THE HIP | EXERCISE FOCUSHED ON THE PECTORAL GIRDLE |
| FAMILIARIZA-TION PERIOD (WEEKS 1-2) | Neck and dorsal spine flexion | Hip extension | Scapula abduction |
|  | 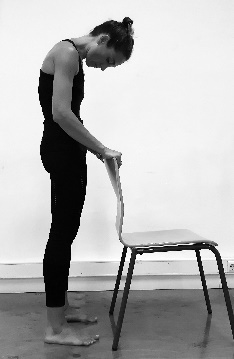 | 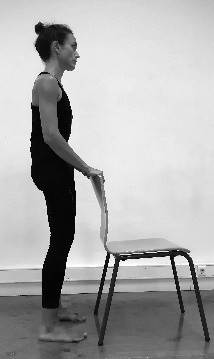 | 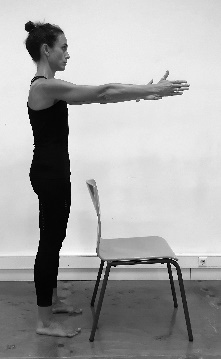 |
| MESOCYCLE 1 (WEEKS 3-6) | Standing spine twist | Standing hip extension | Windmill arms seated on a chair |
|  | 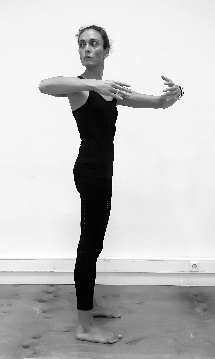 | 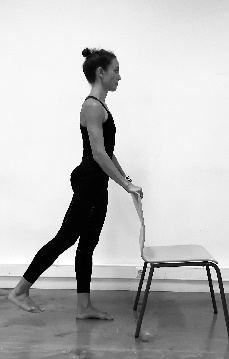 | 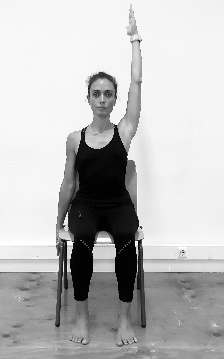 |
| MESOCYCLE 2 (WEEKS 7-10) | Supine curl ups (chi ball) | Side leg lifts | Supine up shoulders  (elastic band) |
|  | 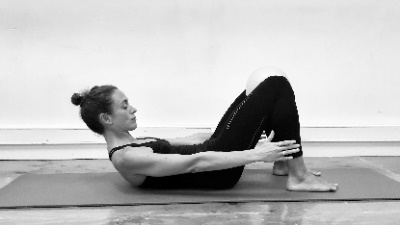 | 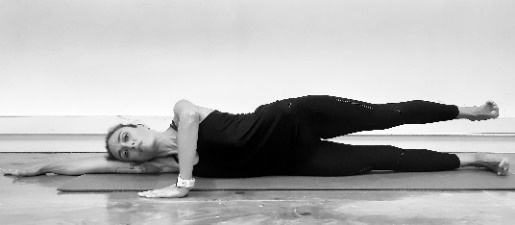 | 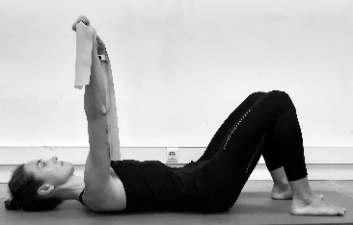 |
| MESOCYCLE 3 (WEEKS 11-14) | The bridge | Side leg lifts (chi ball) | Supine windmill arms  (elastic band) |
|  | 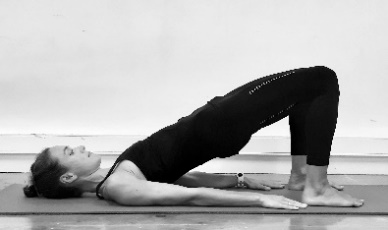 | 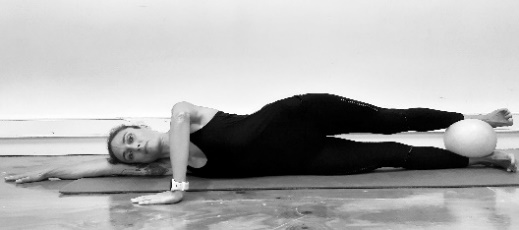 | 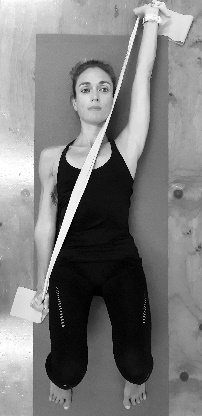 |
| MESOCYCLE 4 (WEEKS 15-18) | Assisted roll up  (elastic band) | Femur arcs and windmill arms  (elastic band) | Curl ups and shoulder abduction (elastic band) |
|  | 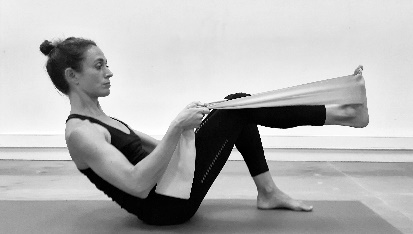 | 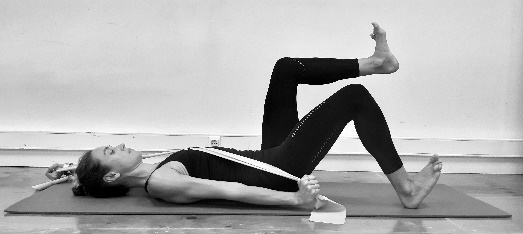 | 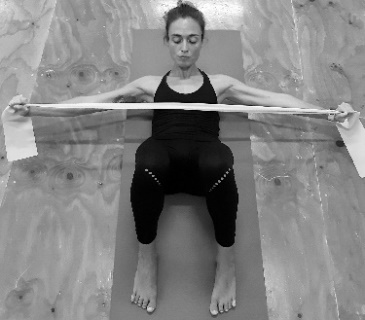 |
