# Supplementary material for: Pilates versus resistance training on trunk strength and balance adaptations in older women: a randomized controlled trial
Source: PeerJ. 2019 Nov 14;7:e7948. doi: 10.7717/peerj.7948 (PMC6859004; doi:10.7717/peerj.7948)
Supplement: Supplemental Information 4 [file peerj-07-7948-s004.docx]

**Pilates Versus Resistance Training on Trunk Strength and Balance Adaptations in Older Women: A Randomised Controlled Trial**

María Carrasco-Poyatos^1^, Domingo J. Ramos-Campo^2^, Jacobo A. Rubio-Arias^2^

^1^ Department of Education. Health and Public Administration Research Center, Universidad de Almería, La Cañada de San Urbano, Almería, Spain

^2^ Department of Physical Activity and Sport Sciences. UCAM Research Centre for High Performance Sport, Universidad Católica San Antonio. La Ñora, Murcia, Spain.

Corresponding Author:

María Carrasco-Poyatos^1^

Carretera Sacramento s/n., La Cañada de San Urbano, Almería, 04120, Spain.

Email address: [carrasco@ual.es](mailto:carrasco@ual.es)

**Abstract**

Background. The neuromuscular decline impact in old women functional independence is determining the necessity to implement new strategies focused on trunk strength training and postural stability maintenance to promote healthy ageing.

Objectives. To define whether Pilates or resistance training is better at improving a) trunk isometric and isokinetic muscular strength, and b) static and dynamic balance, in older women.

Methods. This was a cluster randomized controlled trial. Physically independent older women (60–80 years) from day centres were randomly allocated to Pilates, Muscular and Control Groups (PG, MG and CG, respectively) using block randomization method. Only the research staff performing the assessment and statistical analysis were blinded. Exercise groups trained twice a week (1 hour per session) for 18 weeks in a moderate-to-vigorous intensity. Trunk strength (primary outcome): trunk and hip isometric and hip isokinetic muscular strength (Biodex System III Pro Isokinetic Dynamometer), alongside one leg static balance (portable force platform Kistler 9286AA) and dynamic balance (Timed Up and Go) were assessed.

Results. Sixty participants were randomized (PG, n=20; MG, n=20; CG, n=20) and forty-nine completed the trial (PG, n=16; MG, n=19; CG, n=14). Regarding hip isometric extension strength, PG was statistically better than CG (*P* = 0.004). There were no differences between groups regarding isokinetic strength or balance. Intra-group comparisons showed significant improvements (*P* < 0.05) in the dynamic balance and trunk and hip isometric extension strength for PG and MG, whereas every hip isokinetic measurement was improved in MG. Exercise programs did not produce any adverse event.

Conclusions. The Pilates training program was more effective for improving isometric hip and trunk extension strength, while the Muscular training program generated greater benefits on trunk and hip isokinetic strength. Moreover, both training programmes showed moderate effects for the Timed Up and Go.

The trial was registered at ClinicalTrials.gov (identifier: NCT02506491).

Funding: This work was supported by the San Antonio Catholic University (PMAFI/24/14).

**RESEARCH PROTOCOL**

**1. PARTICIPANTS**

- 80 older women were invited to participate in the study.
- They were recruited from old people day centers from Murcia (Spain).
- A general medical evaluation was accomplished.
- In total 60 women were enrolled in the study and randomly distributed into Pilates group (PG), Muscular group (MG) and Control group (CG).

**2. INCLUSION CRITERIA**

- Women 60–80 years old.
- Physically able to develop the basic and instrumental activities of daily living.
- Without cognitive impairment or diseases that can affect musculoskeletal or cardiovascular systems

**3. EXCLUSION CRITERIA**

- Women who were currently participating or had previously participated in a structured Pilates or resistance training exercise program in the past 3 months.
- Women with a visual or auditory impairment not corrected with glasses or a hearing aid.
- Not maintaining at least 80% (29 sessions) compliance with the exercise session.

**4. RANDOMIZATION**

- A block randomization method was used according to allocation of the specialized senior centres.
- Block size was determined by the research staff according to the statistical power provided.
- Blocks were chosen randomly. A randomization sequence was created using Excel 2016 (Microsoft, Redmond, WA, USA) with a 1:1 allocation using a random number table.
- Only the research staff performing the assessment and statistical analysis were blinded to the exercise group assignment.

**5. INTERVENTION**

- Participants allocated to PG or MG were required to train twice a week (1 hour per session) for 18 weeks from January to May (2016).
- Women assigned to CG were encouraged to maintain their normal physical activity habits.
- The exercise programs were conducted by the same accredited exercise expert who was certified in personal training and Pilates.
- The programs were divided into a 2-week familiarization period and four 4-week mesocycles that were designed to be progressively more challenging.
- The sessions were given in three phases: (1) the warm-up, (2) the Pilates or resistance training exercise programs and (3) the cool-down.
- Intensity was controlled using the OMNI-Resistance Exercise Scale of perceived exertion, beginning at a moderate intensity (6–7 points) and finishing at a moderate-to-vigorous intensity (8–9 points).
- The Pilates and resistance training exercise programs were focused on stimulating the muscles in a dynamic and static way. Balance was an essential part of the standing exercises. The Pilates exercise program also incorporated the principles of Pilates.

**6. ASSESSMENT**

- The primary outcome measures were trunk and hip isometric and isokinetic strength.
- The secondary outcome was balance.

**6.1. Trunk and hip isometric and isokinetic strength**

- Biodex System III Pro Isokinetic Dynamometer (Biodex Medical System, NY, USA) was used.
- A warm up on a bicycle ergometer for 5 minutes followed by 5 minutes of stretching exercises was carried out.
- For hip assessment, participants lay supine on the dynamometer chair. The chest, pelvis and non-tested thigh were fixed to the dynamometer chair. The rotation axis was set at the level of the femoral joint. For isokinetic test, the range of movement in the tested hip was adapted to the flexion capacity of each participant. For isometric test, the hip was fixed at 90º flexion.
- For trunk assessment, the participant trunk was fixed at 90º flexion. The rotation axis was set at the level of L5–S1.
- For isokinetic testing, participants executed five concentric-concentric contractions at low (60º/s) and high (120º/s) velocity with 2 minutes of rest in-between. Prior to the test, a familiarization set of five submaximal repetitions was performed at each protocol speed.
- For isometric testing, five sustained maximal voluntary isometric flexion and extension contractions of 5 seconds were executed with a 5-second rest period in-between.

**6.2. Balance**

- Static balance was measured with a portable force platform (Kistler 9286AA. Kistler instrumente AG, Winterthur, Switzerland). Participants were barefoot and maintained an upright position with their hands hanging loosely down and their eyes open. Their gaze was fixed on a mark at eye level. Right and left single support was performed. Measurements were conducted in three 30-second trials with 1 minute of rest in-between.
- Dynamic balance was assessed using the 3-metre walk Timed Up and Go (TUG) test.

**7. STATISTICAL METHODS**

- Statistical analyses were conducted using SPSS Statistics 23.0 (Armonk, NY, USA).
- Intention-to-treat analysis using last observation for missing data was conducted.
- To compare variables after the intervention, ANCOVA analyses with baseline values included as co-variables were used.
- As additional analyses, Student’s *t*-test for dependent samples was used.
- The effect size was calculated.
- The level of significance was set to *P* < 0.05.
